# Levo-Stepholidine as a Potential Cognitive Enhancer: Insights into Executive Function and Memory Improvements

**DOI:** 10.3390/biomedicines12122680

**Published:** 2024-11-25

**Authors:** Zhengwei Hu, Xueqing Zhu, Yirui Liang, Yuqiu Zhang, Ping Zheng, Xuehan Zhang

**Affiliations:** State Key Laboratory of Medical Neurobiology, MOE Frontiers Center for Brain Science, Institutes of Brain Science, Fudan University, Shanghai 200032, China

**Keywords:** Chinese herb, antipsychotic agents, D1 receptor agonist, D2 receptor antagonist, cognition, executive function, memory, glutamate receptors, prefrontal cortex, Sprague-Dawley rats

## Abstract

Background/Objectives: Levo-Stepholidine (*l*-SPD), a compound extracted from Chinese herbs, has the potential to treat psychotic disorders where cognitive deficits are a critical challenge. *L*-SPD displays a D1R agonism/D2R antagonism pharmacological profile, and its effect on cognitive function is still vague and lacks comprehensive study. Here, we investigated the impact of l-SPD on two core indexes of executive function, working memory and response inhibition, and learning and memory. Methods: Using a delayed alternation T-maze task (DAT), we investigated the impact of *l*-SPD on working memory, evaluated its effect on response inhibition using the stop-signal task (SST), and assessed the impact on learning and memory using trace fear conditioning in Sprague-Dawley rats. We further evaluated its effects on prefrontal glutamate receptor expression using western blot. Results: Rats receiving *l*-SPD made fewer errors in the T-maze, exhibited faster stop action in response to the stop signal, and showed longer-lasting memory retention. Molecular mechanism investigations reveal that *l*-SPD upregulates the expression of prefrontal glutamate receptors. These results demonstrate that *l*-SPD improves executive function and memory. Conclusions: Here, we show the enhancement effect of l-SPD on cognitive function, which provides essential implicants for the treatment of cognitive deficits, which is a critical unmet need in psychiatric care.

## 1. Introduction

Cognitive function is a mental process which involves several intellectual abilities, such as perception, reasoning, and remembering, while cognitive impairment is the malfunction of cognition or intellectual skills [1]. Cognitive deficits are a core feature of psychotic disorders clinically observed in a range of psychotic disorders, especially in schizophrenia [2]. Although the diagnosis of schizophrenia is based on the presence of positive symptoms, such as hallucinations and delusions, and negative symptoms, such as avolition, alogia, and expressive deficits, cognitive dysfunction is regarded as a core component of the disorder [3,4]. Cognitive dysfunction often precedes the onset of psychosis by several years and is an essential factor closely associated with functional outcomes in schizophrenia [5,6]. Despite this fact, current treatment strategies largely fail to alleviate the cognitive deficits in patients with psychotic disorders, such as schizophrenia, bipolar disorder, and psychotic depression [7].

Patients with psychotic disorders experience broad cognitive deficits. These deficits include attention, memory, reasoning, and executive function, which also impair medication adherence in patients with schizophrenia, resulting in an increased risk for relapse of psychosis, persistent symptoms, and suicide attempts [8,9,10,11]. These deficits negatively impact functional outcomes, contributing to the disabling nature of these disorders. Therefore, there has long been a search for effective treatment strategies for cognitive deficits in patients with psychotic disorders.

Executive function, also called cognitive control, is a higher-order cognitive function needed for adaptive goal-directed behaviors and is significantly impaired in most neuropsychiatric disorders [12,13]. It is generally agreed that working memory, response inhibition, and cognitive flexibility are the three core executive functions [14]. Deficits in executive function have long been thought to be one of the hallmark cognitive characteristics of schizophrenia. Among the executive functions that have been well studied, working memory, the ability to transiently maintain and manipulate a limited amount of information to guide thought or behavior [15], is robustly impaired in patients with schizophrenia [16]. Some studies indicate that working memory deficits might be a core contributing factor to disturbances in other cognitive domains in schizophrenia [17,18,19]. Further, executive control processes allow individuals to flexibly and dynamically adjust their performance in response to changing environmental demands and changing internal goal states, such as response inhibition, which refers to the ability to suppress both dominant and already-activated responses, which is impaired in schizophrenia [20].

The prefrontal cortex (PFC) plays a vital role in executive function, including working memory, inhibitory control, flexibility, and attention regulation, and is essential for learning and memory [12,21]. These cognitive functions are heavily influenced by dopaminergic synapses. Dopamine can signal through multiple receptors, such as D1-like (D1 and D5) or D2-like (D2, D3, and D4) [22]. The importance of D1 receptors (D1Rs) and D2 receptors (D2Rs) signaling is highlighted by their requirement for PFC cognitive functions [23,24,25] and their disruption in many psychotic disorders [26]. Besides dopamine, glutamate is essential to PFC cognitive functions through its receptors, such as the N-methyl-D-aspartic acid receptor (NMDAR) and AMPA receptor (AMPAR) [27]. Hypofunction of these glutamate receptors is a critical pathogenic factor of schizophrenia [28] and is observed in psychotic disorders [29]. The glutamate and dopamine hypotheses are leading theories of the pathophysiology of schizophrenia. Moreover, the dopamine receptor signalings could regulate NMDAR and AMPAR expression and trafficking in PFC [30,31].

*l*evo-Stepholidine (*l*-SPD) is an alkaloid of the tetrahydroberberine class, extracted from the Chinese medicinal plant *Stephania intermedia*. It displays a significant binding affinity for D1-like receptors and a moderate affinity for D2-like receptors [32,33,34,35]. Preclinical and clinical trials have indicated that *l*-SPD can alleviate both positive and negative symptoms of schizophrenia. These effects are achieved through D2 receptor antagonism in the nucleus accumbens (NAc) and D1 receptor activation in PFC, respectively [32,36]. Therefore, *l*-SPD, as an antipsychotic medication with a D1R agonism/D2R antagonism pharmacological profile, should, in theory, control psychosis and treat cognitive symptoms by acting on the cortical dopamine system. Several articles describe its impact on cognitive function, but it has not been systematically evaluated [37,38,39,40,41].

In the present study, we evaluated the effects of *l*-SPD on executive function and memory function using multiple behavior paradigms. We further illustrated its impact on glutamate receptor expression in PFC using western blot. Working memory and response inhibition are the core of executive function. The PFC guides behavior and thought through working memory [42]. Response inhibition is a fundamental building block for more complex PFC cognitive operations [43]. The delayed alternative T-maze task was used to test rodent working memory, whose contents can represent a recently visited place that is temporarily held in mind to guide forthcoming behavior [42,44]. Animals must remember the T-maze arm they visited in the last trial while waiting for the subsequent trial. Then, they can correctly decide to enter into the baited arm and get rewards in the following trial. Therefore, the percentage of correct choices to get into the arm indexes working memory [45]. The stop-signal task (SST) was used to detect response inhibition in the clinic and laboratory [46,47], where the reaction time in response to the stop signal was estimated using the independent race model, which describes performance in the SST as a race between two independent internal processes, keep going or stop [48]. The stop-signal reaction time, which characterizes response inhibition ability in individuals, is independent of the training strength. We used the trace fear conditioning paradigm to investigate impact of l-SPD on learning and memory. Trace fear conditioning is a type of classical associative learning characterized by a temporal gap between the presentation of the conditioned stimulus (CS) and the unconditioned stimulus (US). The PFC is involved in acquiring and storing trace fear memory [49,50]. Successful association in this form of conditioning relies on activity within the PFC [49]. The freezing behavior during the temporal gap in absence of CS represents trace fear memory.

The present study found that *l*-SPD improved executive function, enhanced memory, and upregulated glutamate receptor expression in PFC. These results provide a clue for clinical treatment of cognitive dysfunction in psychotic disorders.

## 2. Materials and Methods

To address the impact of *l*-SPD on executive function and memory, we performed multiple experiments to investigate the effects of *l*-SPD on working memory using the delayed alternative task in T-maze (DAT) [45,51], illuminated its effect on response inhibition using the stop-signal task (SST) [47,52], and assessed the impact on learning and memory using the trace fear conditioning paradigm in Sprague-Dawley rats [50,53], as well as evaluated its effects on prefrontal glutamate receptor expression using western blot [54,55].

### 2.1. Subjects

Male Sprague-Dawley rats (180–200 g) were purchased from SLACCA (Shanghai, China). Rats were housed in plastic cages (3–4 per cage) and placed on a 12-h light/dark cycle at 23 ± 1 °C room temperature. Animals had unrestricted access to food and water throughout the study. All experimental protocols received approval from the Ethical Committee of Animal Experiments at Fudan University School of Basic Medical Sciences (Shanghai, China) and complied with the National Institutes of Health Guide for the Care and Use of Experimental Animals (1996). All efforts were made to minimize the number of animals used and their suffering.

### 2.2. Behavioral Experiments

**Scheme 1:**
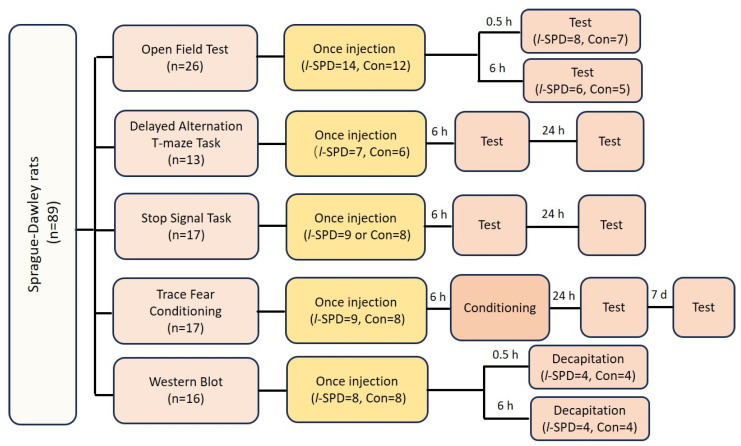
The diagram for the experiment design.

L-SPD (5 mg/kg) and equal volume vehicle (60% DMSO) were delivered by intraperitoneal injection. Each rat received one injection and was subject to one experiment. The following scheme shows the diagram for the experiment design (Scheme 1).

### 2.3. Open Field Test

The open-field chamber (60 × 60 × 50 cm) consists of opaque grey PVC panels. Rats were gently put at the center of the chamber and allowed to explore the arena freely for 15 min. The total distance traveled in the chamber was analyzed using the EthoVision XT Behaviour Observation Record Analysis System (Noldus, Wageningen, The Netherlands).

### 2.4. The Delayed Alternative Task in T-Maze

The delayed alternative task in the T-maze (DAT) was conducted according to our previous study [45]. The apparatus consists of one start arm (50 cm in length, 10 cm in width, and 20 cm in height) and two goal arms (40 cm in length, 10 cm in width, and 20 cm in height). In the habituation stage, rats were subjected to a restricted diet and maintained at approximately 90% of their original weight for one week with a regular water supply. Then, they were habituated to a T-maze until they voluntarily ate a piece of peanut at the end of each arm and returned to the home cage for two consecutive days. Throughout the training period, daily sessions included one initial trial followed by ten formal trials. Each trial started with the sliding door being opened, allowing the rats to explore the maze. In the initial trial, both arms were baited, and the rats were rewarded for visiting either arm. At each formal trial, the rat was allowed to find and eat a piece of peanut in the opposite goal arm chosen previously; then, the rat was guided back into the start arm, and the sliding door was closed. The apparatus were wiped using 10% alcohol to remove odor after each trial. At the end of the 10-s interval (delay), the rat was allowed to explore the two goal arms. If the rat entered the opposite goal arm, a reward was given, and a correct choice was scored; if the rat entered the goal arm chosen previously, an incorrect choice was scored. The percentage of correct choices for each rat was calculated.

The testing began the next day when the correct rate was stable at 60–80% on two consecutive days. The rats were given *l*-SPD (5 mg/kg i.p.) 6 h before the testing. The testing process is the same as the training. If the rat chooses the wrong arm, keep the baited arm to remain baited until it appears, giving the rat a chance to change their choice. We defined a win-shift failure as the rat entering the arm that had been the correct choice in the previous trial. In contrast, a lose-shift failure was defined as the rat repeatedly entering an arm that had been the incorrect choice in the last trial [45]. The animals were re-tested one day after testing.

### 2.5. The Stop-Signal Task

A rat nose-poking stop-signal task (SST) was conducted according to our previous study [47]. A pure tone auditory stimulus, approximately 4 kHz at 60 dB, was used as a stop signal to indicate to the rat to cease poking behavior. In total, 20% of the trials were stop trials and presented randomly. When the tone is delivered, the rat should immediately cancel poking to the action port. The rat could get water in the reward port by successfully withholding this process. Any wrong trial resulted in a 5-s punishment with all lights off and no water delivery. When the rats became proficient at the task, achieving both go and stop correct ratios of over 90% during the session, they could proceed to further testing.

The testing phase consisted of two stages: one adaptive stage of 20 trials and three testing stages of 100 trials each. In the adaptive stage, only go trials were conducted. During the subsequent testing stages, the stop signal was introduced and delivered at variable delays, known as stop signal delays (SSDs). A dynamic adjustment of the SSD was used to achieve a 50% success rate for the stop trials. During the adaptive stage, the initial SSD was set to the median of the sorted Go response time (Go RT) minus 200 ms. In the subsequent testing stages, each block consisted of 100 trials, including 20 stop trials and 80 go trials, with all stop trials randomly interleaved within the block. The SSD was adjusted after every stop trial based on the outcome: if the stop trial was successful, the SSD was increased by 50 ms for the subsequent stop trial; if the stop trial failed, the SSD was decreased by 50 ms.

According to the independent-race model, which describes performance in the stop-signal task as a race between two independent internal processes [48], the stop-signal reaction time (SSRT) can be used to measure a rat’s response inhibition ability. A lower SSRT indicates better inhibition of inappropriate responses, suggesting that the rat can quickly stop its behavior in response to the stop signal. Conversely, a higher SSRT demonstrates that the rat finds it more difficult to inhibit inappropriate behavior, as responding to the stop signal takes longer. The SSRT was calculated separately for each block and then averaged to provide a final estimate for each rat in one testing session.

The stop-signal reaction time (SSRT) was estimated according to the independent-race model, whereby performance in the stop-signal task is modeled as a race between two independent internal processes [48]. The SSRT was calculated for each block separately and then averaged to yield a final estimate for each rat in one testing session.

### 2.6. Trace Fear Conditioning

Trace fear conditioning was conducted according to our previous study [53]. Briefly, rats were habituated for approximately 180 s in the training box. The entire session consisted of seven trials over a 16-min period. Each conditioning trial began with a 10-s pure tone as the conditioning stimulus (CS). Following a 40-s trace period, an electric foot shock (0.5 mA, 0.5 s) was administered as the unconditioned stimulus (US). The inter-trial intervals were randomly set between 1 and 3 min. Freezing behavior during the 40-s trace periods was measured for each animal (Med Associates Inc., Georgia, VT, USA). The retention of the CS-US association was measured by monitoring freezing behavior one day after conditioning. The animals were gently placed in a novel context and allowed a 120-s habituation period. Only a 10-s tone, the same as the conditioning (2.2 kHz, 80 dB), was delivered, but no foot shock followed. Seven days later, the retention of the CS-US association was re-measured in a different context from one-day testing. Three of the four retention trials were used for further analysis. The percentage of freezing time was measured during the trace period, which is the time following the tone (40 s after the tone).

### 2.7. Western Blot

The rats were decapitated following an overdose of isoflurane. Then, brains were rapidly removed and put into the 0 °C artificial cerebrospinal fluid. Bilateral PFC tissues were quickly dissected, frozen in liquid nitrogen, and stored at −80 °C until tissue lysis. The following steps were conducted according to our previous study [54]. We used the Membrane Protein Extraction Kit (K268-50, BioVision, Palo Alto, CA, USA) to isolate membrane protein according to the instructions. Briefly, the PFC tissues at −80 °C were taken out, homogenization buffer was added, and the tissues were sonicated twice with ultrasonic cell crushing apparatus, each time for 10 s. The homogenates in 1.5 mL microcentrifuge tubes were centrifuged at 700× *g* for 10 min at 4 °C. The supernatant was collected and centrifuged at 10,000× *g* for 30 min at 4 °C. The sediments were resuspended with the Upper Phase Solution and blended with the Lower Phase Solution. The mixture was centrifuged at 1000× *g* for 5 min. The upper phase was collected and diluted with a 5-fold volume of water. The solution was centrifuged at 10,000× *g* for 30 min at 4 °C. The sediments were dissolved in Triton X-100 in PBS. The protein concentration was determined with the Pierce™ BCA Protein Assay Kit (Thermo Fisher Scientific, Waltham, MA, USA), and normalized to 1.0 μg/μL per sample. The samples were boiled in SDS-PAGE sample loading buffer (Beyotime, Shanghai, China), separated by Sodium Dodecyl Sulfate-Polyacrylamide Gel Electrophoresis (SDS-PAGE) with an 8% polyacrylamide gel and transferred to PVDF membranes (Roche) by electroblotting (Bio-Rad, Milano, Italy). The PVDF sheets were blocked in QuickBlock™ Blocking Buffer for Western Blot (Beyotime) at 37 °C for 15 min. The blots were incubated with primary antibody diluted in 5% nonfat dry milk in TBST overnight at 4 °C and incubated with secondary antibodies for 1 hr. Signals were finally visualized using enhanced chemiluminescence (Thermo Fischer Scientific), and the blots were exposed onto Alpha FluorChem FC3 (proteinSimple) and analyzed using AlphaView SA. For each rat, the data are presented as the mean from at least three separate experiments.

The antibodies used in the study included anti-GluR1 (1:600; Abcam, Cambridge, UK), anti-GluR2 (1:800; Abcam), anti-GluN2A (1:500; Millipore, Billerica, MA, USA), anti-GluN2B (1:800; Millipore), anti-PSD95 (1:4000; Abcam), peroxidase-conjugate goat anti-mouse or goat anti-rabbit antibodies (1:5000; Jackson, Philadelphia, PA, USA).

### 2.8. Chemicals

*l*-SPD was dissolved into 60% DMSO. *l*-SPD was purchased from Ji Qi Pharmaceutical Company (Guilin, China)

### 2.9. Data Analysis

The data were shown as the mean ± SEM. Statistical analysis of the drug-induced difference was compared using the paired student’s *t*-test in DAT and using the student’s *t*-test in the open field test, trace fear memory test, and western blot when data passed the normality test; otherwise, using the rank sum test. Win-shift failure was compared using the Mann-Whitney U-test. The paired student’s *t*-test was used to statistically analyze the drug-induced differences in the stop-signal task (SST). A non-parametric permutation test was employed to analyze the normalized stop-signal reaction times (SSRTs). Two-way ANOVA was used to analyze the drug-induced difference in trace fear memory acquisition. *p* < 0.05 was indicated significance. tatistical comparisons were conducted using SigmaPlot 10.0 (Systat Soft Inc., San Jose, CA, USA) and R. All graphs were plotted using SigmaPlot 10.0.

## 3. Results

### 3.1. Effect of l-SPD on Spontaneous Activity of Rats

**Figure 1:**
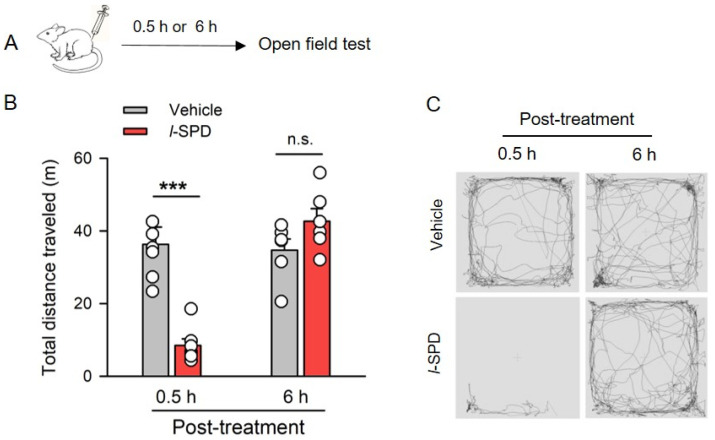
Effect of *l*-SPD on spontaneous activity of rats. (**A**) Scheme of the experimental procedure. (**B**) The total distance traveled in the open field chamber of animals tested at 0.5 h or 6 h post systemic administrations of *l*-SPD (5 mg/kg) versus vehicle. *** *p* < 0.001, n.s. no significance, student’s *t*-test. n = 6–8. (**C**) Demo traces traveled in the open field chamber of animals.

To examine whether systemic administration of *l*-SPD affects the spontaneous activity of rats, we intraperitoneally injected *l*-SPD (5 mg/kg) or vehicle into rats 0.5 h or 6 h before the open field test (OFT) (Figure 1A). As shown in Figure 1B, animals that received *l-*SPD (8.5 ± 1.7 m) traveled far shorter distances in the chamber than vehicle-treated animals (36.3 ± 4.5 m) when animals were examined at 0.5 h after intraperitoneal injection (*p* < 0.001 for *l*-SPD vs. vehicle, student’s *t*-test; n = 7–8; Figure 1B). While *l*-SPD-treated animals (42.7 ± 3.1 m) traveled a comparable distance with vehicle controls (34.7 ± 2.9 m) examined at 6 h after injection (*p* > 0.05; n = 6; Figure 1B).

These results suggested that *l*-SPD dramatically reduced spontaneous activity 0.5 h after injection but spared spontaneous activity 6 h after injection in rats (Figure 1C). Therefore, behavioral tests were carried out 6 h after *l*-SPD administrations in the following experiments to avoid abnormal locomotor function affecting behavior parameter measurement.

### 3.2. L-SPD Enhances the Working Memory in Delayed Alternative T-Maze Task

**Figure 2:**
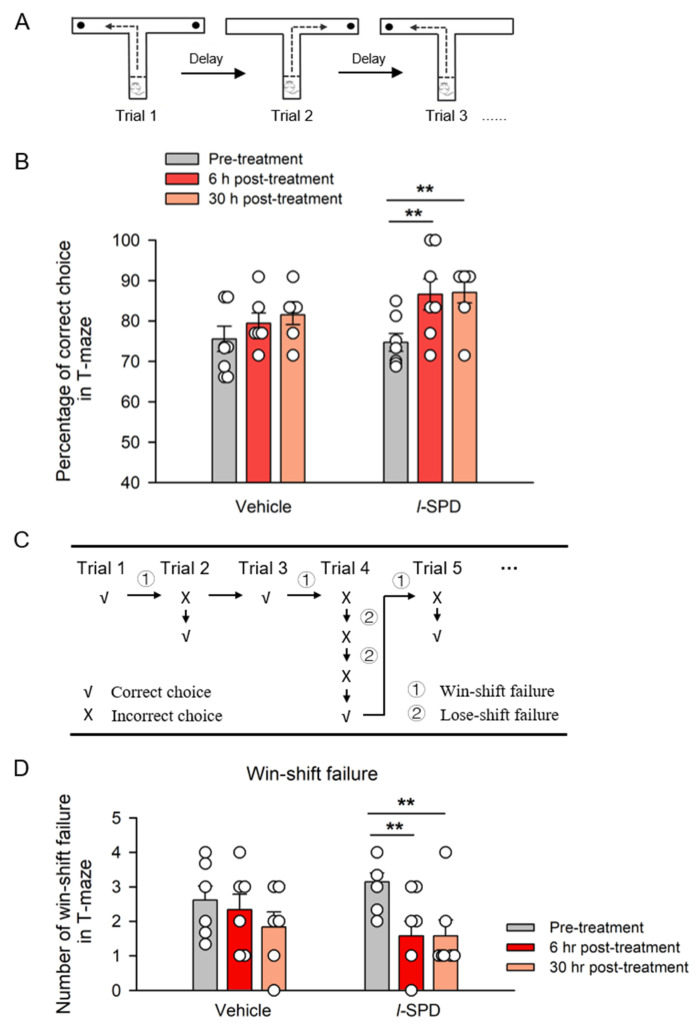
*l*-SPD enhances the working memory of rats in a delayed alternative task in T-maze. (**A**) Illustration of the delayed alternative T-maze task (DAT task). (**B**) Performance in DAT task before (baseline), 6 h post, and 30 h post-injection of *l*-SPD versus vehicle. (**C**) Schematic diagram of win-shift failure in DAT task. (**D**) The number of win-shift failures in the DAT task before (baseline), 6 h post, and 30 h post-injection of *l*-SPD versus vehicle. ** *p* < 0.01 versus baseline, paired *t*-test. n = 6–7.

To address the effect of *l*-SPD on working memory, we trained rats on a delayed alternative task in a T-maze (DAT). In this behavioral paradigm, to perform the task correctly, rats had to remember which arm had visited in the previous trial during the delay and select the opposite arm (Figure 2A). When rats performed the DAT task at ~60–70% accuracy on two or three consecutive days of testing, rats received intraperitoneal injections of *l*-SPD or vehicle. We tested the effect of *l*-SPD on performance in DAT with a 10-s delay 6 h post-injection (6 h post-treatment) and re-tested 24 h later (30 h post-treatment). As shown in Figure 2B, the *l*-SPD-treated animals made fewer error choices. That is, the percentage of correct choice increased from 74.7 ± 2.2% (pre-treatment) to 86.6 ± 3.8% 6 h post-treatment (Figure 2B, *p* < 0.01 for 6 h post- versus pre-treatment, paired *t*-test; *l*-SPD, n = 7) and kept 87.07 ± 2.6% tested 24 h later (Figure 2B, *p* < 0.01 for 30 h post- versus pre-treatment). The performance in DAT was comparable between 6 h and 30 h of *l*-SPD injection (*p* > 0.05, student’s *t*-test). At the same time, animals who received vehicles showed no change in their performance compared with their pre-treatment tested at 6 h post-treatment (Figure 2B, *p* > 0.05; vehicle, n = 6) and re-tested 24 h later (Figure 2B, *p* > 0.05).

In the study, a correction procedure was implemented for cases where rats made a consecutive incorrect choice; the same arm was baited again to give the rats an opportunity to change their selection. Rats were given as many correction trials as needed, meaning the same arm was repeatedly baited until they made the correct choice. Figure 2C illustrates two types of performance errors: a win-shift failure, where rats did not change their choice after selecting the correct arm in the previous trial, and a lose-shift failure, where they repeated an incorrect choice from the last trial. Win-shift failure indicates a deficit in working memory, while lose-shift failure reflects a deficit in error-correction ability. As shown in Figure 2D, analysis of Win-shift failure number revealed that the animals that received l-SPD showed a better ability in using the Win-shift strategy tested at 6 h post-treatment (*p* < 0.01 for 6 h post- versus pre-treatment) and re-tested 24 h later (*p* < 0.01 for 30 h post- versus pre-treatment) compared with their baseline performance without treatment (Figure 2D). Animals that received vehicles showed unchanged abilities in using the Win-Shift strategy (Figure 2D, *p* > 0.05). Animals made very few lose-shift failures, which did not change after *l*-SPD.

These data indicated that systemic administration of *l*-SPD improves the working memory of rats.

### 3.3. l-SPD Enhances Response Inhibition in the Stop-Signal Task

**Figure 3:**
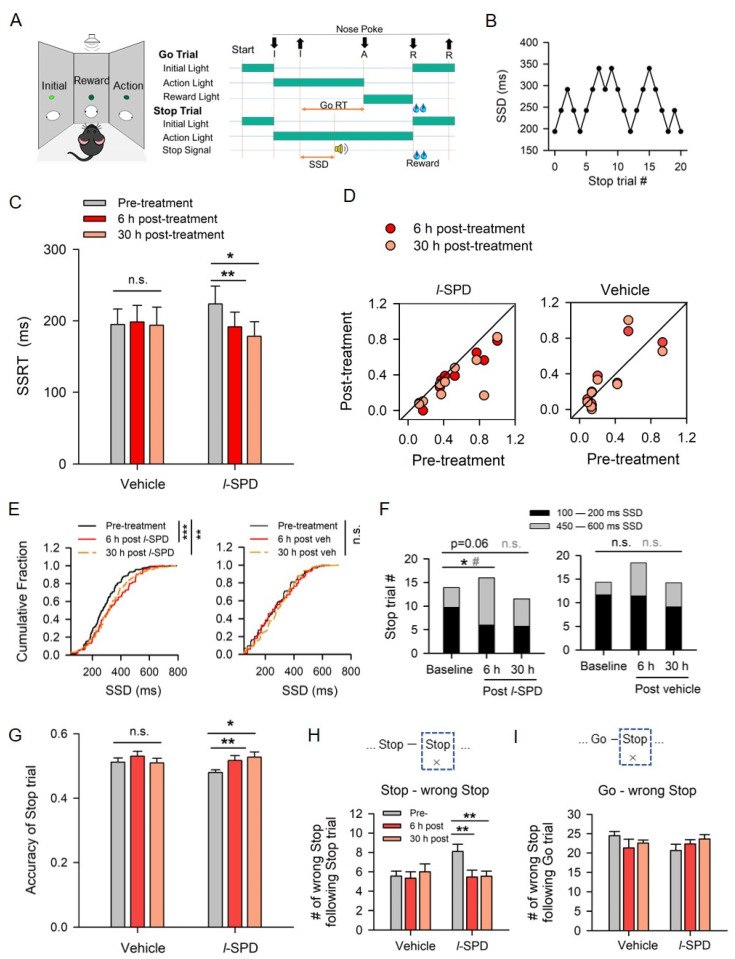
*l*-SPD enhances stop-signal response inhibition of rats. (**A**) Configuration of the operant chamber consists of three snout ports and three lights (green circles). A nose poke into any port is detected by breaking the infra beam (*left panel*). The stop-signal task events in go trials and stop trials show the sequence of animal action (*right panel*). (**B**) Dynamic changed stop-signal delay (SSD) in one session. (**C**,**D**) Stop-signal reaction times (SSRTs) of animals tested before systemic administrations (pre-treatment), 6 h (6 h post), or 30 h (30 h post) after administrations of *l*-SPD and vehicle. * *p* < 0.05, ** *p* < 0.01, n.s. no significance, versus pre-treatment, paired *t*-test (**C**). Normalize SSRTs to pre-treatment of *l*-SPD (*left*) and vehicle (*right*) (**D**). n = 8–9. (**E**,**F**) *l*-SPD prolongs stop-signal delay (SSD). (**E**) Cumulative fraction of all SSD in one testing session tested before treatment (pre-treatment), 6 h, and 30 h post-treatment *l*-SPD (*left*) versus vehicle (*right*), respectively. ** *p* < 0.01, *** *p* < 0.001, n.s. no significance, Kolmogorov-Smirnov test. (**F**) Numbers of stop trials with 100–200 ms (dark bar) and 450–600 ms SSD (grey bar) before (pre-treatment), 6 h post, and 30 h post-treatment of *l*-SPD (*left*) versus vehicle (*right*). Dark star symbol,* *p* < 0.05 for 100–200 ms SSDs, grey # symbol, *p* < 0.05 for 450–600 ms SSDs, n.s. no significance. (**G**) Accuracy of stop trial before treatment (baseline), 6 h and 30 h post-treatment of *l*-SPD versus vehicle. * *p* < 0.05, ** *p* < 0.01, n.s. no significance. (**H**,**I**) Trial numbers of failed stops following stop trial (**H**) or go trial (**I**). Upper panels, diagrams for wrong stop trial following stop trial (**H**) and following go trial (**I**). ** *p* < 0.01.

We used the stop-signal task (SST) to examine response inhibition ability, where the response inhibition was indexed by stop-signal reaction time (SSRT). The shorter SSRT means a better response inhibition ability; the longer SSRT means a worse ability. The training box and schematic of SST were slightly modified according to Logan [48] (Figure 3A). The stop-signal delay (SSD) was dynamically delivered according to the previous status of the stop trial (Figure 3B).

To address the effect of *l*-SPD on response inhibition, we trained rats on SST. When their performance met the criteria, we injected *l*-SPD (5 mg/kg) or vehicle intraperitoneally into rats the following day. SSRTs were detected 6 h after injection (6 h post-treatment) and re-detected 24 h later (30 h post-injection). As shown in Figure 3C, SSRTs decreased from 223.6 ± 24.9 ms (pre-injection) to 191.6 ± 20.7 ms at 6 h (*p* < 0.01) and to 178.5 ± 20.1 ms at 30 h (*p* < 0.05) post-*l*-SPD injection (paired *t*-test; *l*-SPD: n = 9; Figure 3C). Meanwhile, the animals that received the vehicle exhibited comparable SSRTs with their baseline SSRTs at 6 h (*p* > 0.05) and 30 h (*p* > 0.05) post-vehicle injection (student’s *t*-test; vehicle, n = 8; Figure 3C). Figure 3D showed that the normalized SSRTs to pre-treatment at 6 and 30 h after injection were distributed below the diagonal in the *l*-SPD-treated animals (*left*; *p* < 0.001) while distributed on both sides of the diagonal in the vehicle controls (*right*; *p* > 0.05, permutation test; Figure 3D), indicating the decreased effect of *l*-SPD on SSRTs.

We further analyzed the effect of *l*-SPD on stop-signal delay (SSD). The stop signal was delivered after a delay in the experiment. The delay was changing trial by trial, which was determined by the previous status of the stop trial. The longer stop-signal delay (SSD) requires a better response inhibition. As shown in the left panel of Figure 3E, the animals receiving *l*-SPD demonstrated their cumulative fraction of SSDs shifting toward the right tested at 6 h (*p* < 0.001) and 30 h after injection (*p* < 0.01, Kolmogorov-Smirnov test) relative to their baseline SSDs before treatment (Figure 3E, *left*), suggesting that animals fulfilled stop-signal task with longer SSDs after injecting *l*-SPD. The cumulative fraction of SSD in vehicle-treated animals was kept unchanged (*p* > 0.05, Kolmogorov-Smirnov test; Figure 3E, *right*). Analysis of SSDs revealed that the number of stop trials with short SSDs (50–200 ms, dark bars in Figure 3F) significantly decreased by 6 h (*p* < 0.05) and 30 h after *l*-SPD injection (*p* = 0.061), whereas the trial number with longer SSDs (450–600 ms, grey bars in Figure 3F) increased at 6 h post (*p* < 0.05) but not at 30 h post-*l*-SPD injection (*p* > 0.05) (Figure 3F, *left*). Neither the short SSD trial number nor the long SSD trial number changed after vehicle injection (*p* > 0.05) (Figure 3F, *right*). Thus, rats treated with *l*-SPD performed the stop-signal task with longer SSD, indicating that rats receiving administration of *l*-SPD demonstrated better response inhibition.

In addition, *l*-SPD-treated animals demonstrated fewer failures in the stop trial, as shown by increased accuracy in performing the stop trial at 6 h (*p* < 0.01) and 30 h (*p* < 0.05) after injection (student‘s *t*-test; Figure 3G). Moreover, the decreased number of failed stop trials in *l*-SPD-treated animals was only observed in the Stop trial, in which previous trials were Stop trials (*p* < 0.01; Figure 3H) but not Go trials (*p* > 0.05; Figure 3I).

Taken together, *l*-SPD shortened SSRTs and prolonged SSD when rats performed the stop-signal task. These results indicate that *l*-SPD enhances response inhibition.

**Figure 4:**
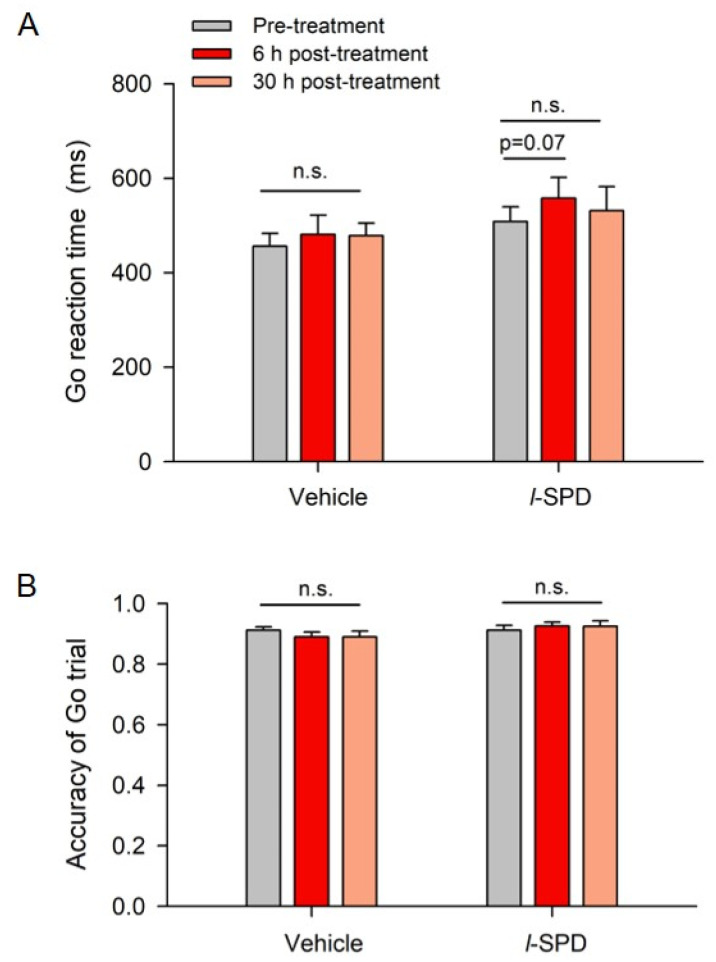
Effects of l-SPD on go trial in stop-signal task. Effect of administration of *l*-SPD (*left panels in A and B*) versus vehicle (*right panels in A and B*) on go reaction time (GoRT) (**A**) and the accuracy of Go trial (**B**). The same data as Figure 3. n.s. no significance.

At the same time, by analyzing the go reaction time (Go RT) and accuracy of the go trial, we examined the effect of *l*-SPD on the go process. As shown in Figure 4, animals did not show a noticeable increase in go reaction time 6 h post (*p* = 0.07) and 30 h (*p* > 0.05) post-*l*-SPD injection (Figure 4A). Neither did the vehicle-treated animals (*p* > 0.05, Figure 4A). Neither *l*-SPD nor the vehicle injection changed the accuracy in performing the go trial (*p* > 0.05; Figure 4B).

These results demonstrated that *l*-SPD enhances response inhibition of rats, and such an enhancement effect lasted to 30 h after *l*-SPD intraperitoneal injection.

### 3.4. l-SPD Enhances Long-Term Memory Retention

**Figure 5:**
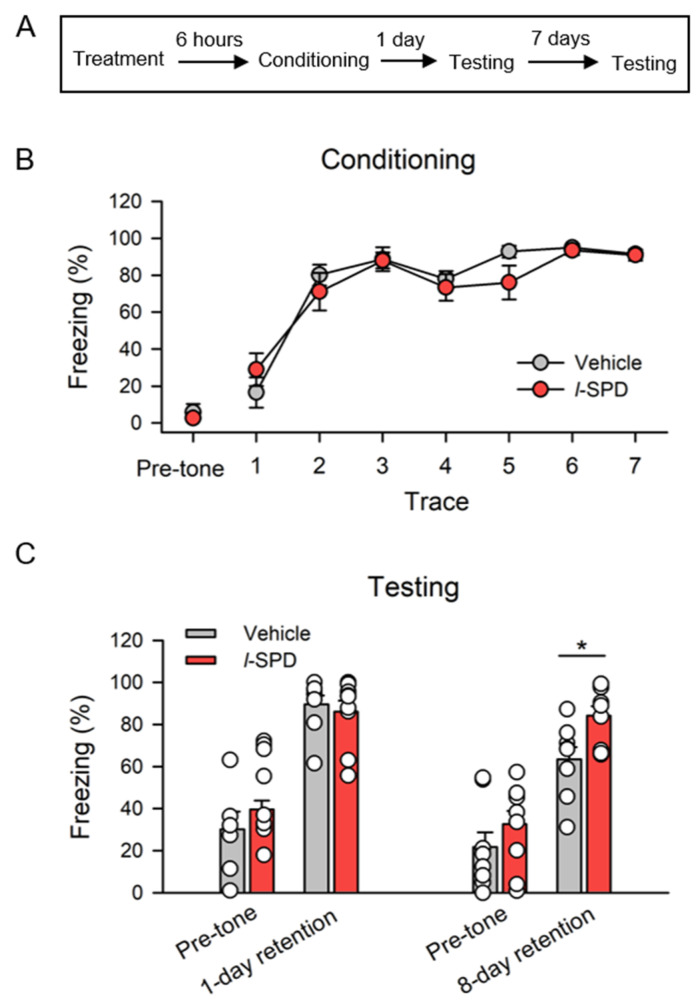
Effects of l-SPD on the acquisition and storage of trace fear memory. (**A**) Scheme of the experimental procedure. (**B**) Freezing behavior of animals treated with *l*-SPD versus vehicle during trace fear conditioning. (**C**) Freezing behavior was examined 1 day and 8 days after conditioning. * *p* < 0.05, student’s *t*-test; n = 8–9.

We used a trace fear conditioning task to assess the effect of *l*-SPD on learning and memory. *l*-SPD (5 mg/kg) or vehicle were intraperitoneally injected into rats 6 h before conditioning. Memory for trace fear was tested one day after conditioning (1-day retention) and re-tested seven days later (8-day retention), respectively (Figure 5A). During the seven CS–US pairings training, all rats showed comparable increased freezing behavior throughout the training sessions (Figure 5B; F_treatment × trace_ (6, 105) = 0.89, *p* = 0.50, two-way ANOVA; *l*-SPD, n = 9; vehicle, n = 8). Memory retention was examined one day after training. All rats displayed similar freezing behavior before the first tone was delivered (*p* > 0.05 for pre-tone, student’s *t*-test) and during the trace period (Figure 5C; *p* > 0.05 for 1-day retention). When trace fear memory retention was re-examined seven days later, the *l*-SPD-treated animals still showed a comparable level of freezing behavior (84.9 ± 4.2%) with 1-day post-conditioning (86.0 ± 5.3%) during the trace period, while the freezing behavior of vehicle controls animals decreased from 89.5 ± 4.2% to 63.3 ± 5.9% (Figure 5C; *p* < 0.05 for *l*-SPD versus vehicle in 8-day retention, student’s *t*-test).

These data demonstrated that *l*-SPD did not affect memory acquisition and 1-day memory retention but enhanced 8-day memory retention.

### 3.5. l-SPD Upregulates NMDA and AMPA Receptor Expression in PFC

**Figure 6:**
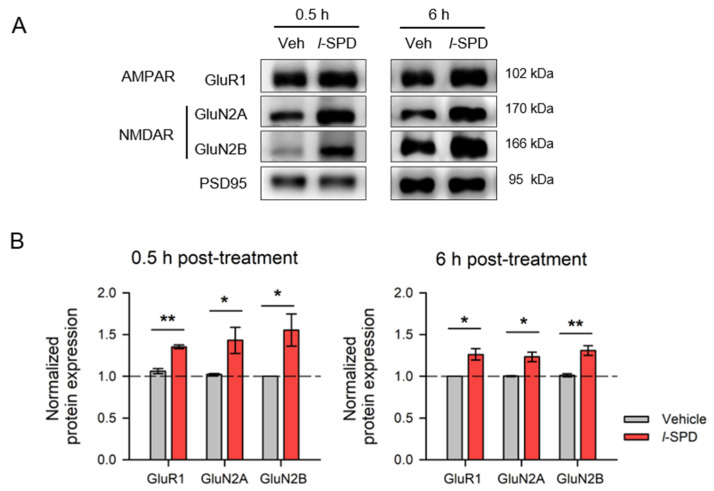
Effect of l-SPD on glutamate receptor expression in PFC. Immunoblot (**A**) and quantification analysis (**B**) of the expressions of AMPAR GluR1 subunits, NMDAR GluN2A, and GluN2B subunits in PFC from animals treated with *l*-SPD versus vehicle examined at 0.5 h post- and 6 h post-treatment (* *p* < 0.05, ** *p* < 0.01; n = 3–4 for each treatment).

Enhancing executive function and memory by *l*-SPD may result from strengthening glutamatergic transmission in PFC [42,56]. To examine the impacts of *l*-SPD on glutamate receptor expression, we measured the amount of NMDAR and AMPAR subunits on the membrane in PFC cells at 0.5 and 6 h after *l*-SPD infusion using western blot, respectively. As shown in Figure 6, animals that received *l*-SPD (5.0 mg/kg) showed a significant increase in GluR1 subunit of AMPAR (*p* < 0.01 for 0.5 h, *p* < 0.05 for 6 h post-injection; n = 4 for each treatment), NR2A (*p* < 0.05 for 0.5 h, *p* < 0.05 for 6 h post-injection; n = 3) and NR2B subunits (*p* < 0.05 for 0.5 h, *p* < 0.01 for 6 h post-injection; n = 3) of NMDAR compared with animals received vehicle (n = 4), respectively.

These data suggested that one intraperitoneal injection of *l*-SPD upregulates glutamate receptor expression on the membrane of PFC cells.

## 4. Discussion

Cognitive deficits are a core feature of psychotic disorders, significantly contributing to the impaired functioning associated with the disorders, and remain unresponsive to current treatments. *L*-SPD, as an antipsychotic medication, has a D1R agonist/D2R antagonist pharmacological profile and relieves symptoms of schizophrenia through D2 receptor inhibition in NAc and D1 receptor activation in PFC. Therefore, in theory, *l*-SPD may treat cognitive symptoms by acting on the cortical dopamine system. However, the effect of *l*-SPD on cognitive functions still lacks comprehensive study. The present study reveals that animals show better working memory, more potent response inhibition, and longer-lasting memory maintenance after receiving *l*-SPD. The beneficial effects of *l*-SPD on these functions may result from its potentiation on glutamatergic transmission in PFC since it upregulated glutamatergic receptor expression in PFC cells. The present study proposes a potential role of *l*-SPD in improving cognitive dysfunction.

*l*-SPD displays a D1R agonist/D2R antagonist pharmacological profile. Many pieces of the literature suggest the beneficial roles of *l*-SPD in animal models by acting on the dopamine system. *l*-SPD reduces *l*-DOPA-induced dyskinesia in the model of Parkinson’s disease [57], prevents methamphetamine-treated mice from memory deficits [40], and rescues memory deficit and synaptic plasticity in models of Alzheimer’s disease and depression via activating dopamine D1 receptor/PKA signaling pathway [37,39]. *l*-SPD has also been implicated in attenuating drug-seeking behavior [41,58,59]. This study used healthy animals instead of model animals since the schizophrenia model animals, such as MK801- and CPP-treated animals [60,61], show severe damage in locomotor function, which impeded evaluating executive function and measuring memory function. Our results showed the enhancement effects of *l*-SPD on executive and memory functions in healthy animals, especially enhanced response inhibition in SST, in which *l*-SPD shortened the stop-signal reaction time, a parameter reflecting inherent ability in healthy animals. These results broadened the knowledge of the beneficial role of l-SPD by acting on the dopamine system.

The present study demonstrates that *l*-SPD enhanced executive function. Animals with systemic administration of *l*-SPD made fewer wrong choices in the DAT task, suggesting an improved effect of *l*-SPD on working memory (Figure 2). In addition, *l*-SPD shortened stop-signal reaction time (SSRT), prolonged the stop-signal delay (SSD), and reduced stop failures in the stop-signal task, suggesting an enhanced effect of *l*-SPD on response inhibition (Figure 3). The enhancement effect of *l*-SPD on working memory may mainly arise from the D1R agonism of *l*-SPD despite studies showing that the D2R antagonism of *l*-SPD exerts negative influences on working memory [62,63,64]. Many studies have demonstrated the role of prefrontal D1R in working memory in nonhuman primates and rodents. In humans, PET imaging studies have implicated D1R in working memory [65,66]. In primates and rodents, local infusions of drugs targeting D1R impair performance on working memory [67,68,69,70,71]. D1R agonists and antagonists can specifically affect neuronal activity related to working memory [67]. At the same time, the enhancement effect of *l*-SPD on response inhibition may link to the D2R antagonism of *l*-SPD in the dorsal striatum [72]. Moreover, it cannot be excluded that *l*-SPD may also enhance cognitive function by promoting dopamine release [73,74].

Besides the direct role of *l*-SPD acting on dopamine receptors in cognitive function, the present study demonstrates that systemic administration of *l*-SPD upregulated the expression of glutamate receptor NMDAR and AMPAR on the cell membrane in PFC (Figure 6), suggesting that *l*-SPD enhances glutamatergic synaptic transmission of PFC neurons. The proper prefrontal NMDAR and AMPAR functions are required for working memory and response inhibition [47,75]. Deficits in glutamatergic transmission impair PFC function [76]. Reducing NR2A mRNA was observed in individuals with schizophrenia [77]. Conversely, increased expression of NMDA receptors in the PFC can enhance learning and memory by facilitating synaptic transmission [78]. *l*-SPD-induced elevation in the expression of glutamate receptors may be the sum of D1R agonism and D2R antagonism in PFC since the PFC glutamate receptor expression upregulating by activating D1R signaling and downregulation by D2R signaling. Thus, *l*-SPD could enhance PFC network activity, thereby enhancing working memory [42] and response inhibition [47]. Moreover, the upregulation of glutamate receptor expression remains 6 h after *l*-SPD administration (Figure 6). This suggests that *l*-SPD may produce a long-term enhancement on glutamatergic synaptic transmission of PFC cells.

The present results demonstrate the enhancement effect of *l*-SPD on trace fear memory. Our data show that systemic administration of *l*-SPD before trace fear conditioning does not affect the acquisition of trace fear memory (Figure 5B), does not affect the memory testing one day after conditioning, but does enhance the long-lasting memory testing eight days after conditioning (Figure 5C), suggesting that *l*-SPD promotes the long-lasting memory storage or prevents the memory from losing along with time passes away. Similarly, these effects may arise directly from PFC D1R activation [79] and indirectly from the potentiation glutamatergic transmission in PFC Synaptic plasticity within the PFC, which is critical for storing trace fear memory [80].

Considering the D2R antagonism of *l*-SPD may lead to sedation, such as other psychotropic medications of D2 antagonism [81], thereby affecting executive and memory function measurement, we conducted the open field test. Our data showed that *l*-SPD reduced locomotion 0.5 h after injection and normal locomotion 6 h after injection (Figure 1). The reduced locomotion 0.5 h after *l*-SPD may relate to the enhancement of GABAergic synaptic transmission in the striatum, in which striatum hypofunction leads to decreased motor activity [82]. We observed a dramatically upregulated expression of GABA receptors in the striatum 0.5 h after *l*-SPD.

The literature reported that doses of 1–30 mg/kg *l*-SPD were used in rodents. We uncovered that 5 mg/kg and 10 mg/kg *l*-SPD enhanced response inhibition in this study. Moreover, no significant difference was observed between 5 mg/kg (Figure 3) and 10 mg/kg *l*-SPD in response inhibition. The open field test showed that rats treated with 5 mg/kg *l*-SPD demonstrated a significant decrease in spontaneous activity after 0.5 h of treatment, while the spontaneous activity returned to normal after 6 h of treatment (Figure 1). Western blot results showed that the expression of PFC glutamate receptors upregulated 0.5 and 6 h after 5 mg/kg of *l*-SPD administration (Figure 6).

Our study demonstrates that *L*-SPD enhances working memory and response inhibition and prolongs memory retention. These effects may arise from the direct role of acting on D1R or D2R and probably result from enhancing neural activity within the PFC through upregulating the glutamate receptor expression in the PFC cell membrane. Our results reveal an essential aspect of *l*-SPD in enhancing PFC functions and, thereby, a potential application for clinically treating PFC hypofunction. However, there are limitations to this research. While we observed significant behavioral changes in healthy animals but not the models of psychotic disorders, further studies are needed to dissect the contribution of D2 antagonism of *l*-SPD in cognitive function improvement. We believe that these studies will provide valuable insights into the role of *l*-SPD in treating cognitive deficits of psychotic disorders, which is a critical unmet need in psychiatric care.

In conclusion, here, we present solid evidence supporting the beneficial roles of *l*-SPD in cognitive and memory functions. This study bridges the gap between pharmacological interventions and cognitive dysfunction in psychotic disorders.

## Data Availability

Data presented in the article is available on reasonable request.
